# Mesenchymal Stromal Cell Therapy for Chronic Lung Allograft Dysfunction: Results of a First‐in‐Man Study

**DOI:** 10.1002/sctm.16-0372

**Published:** 2017-02-01

**Authors:** Daniel C. Chambers, Debra Enever, Sharon Lawrence, Marian J. Sturm, Richard Herrmann, Stephanie Yerkovich, Michael Musk, Peter M.A. Hopkins

**Affiliations:** ^1^ School of Medicine, The University of QueenslandBrisbaneQueenslandAustralia; ^2^ Queensland Lung Transplant Service, The Prince Charles HospitalBrisbaneQueenslandAustralia; ^3^ Western Australian Lung Transplant Program, Fiona Stanley HospitalPerthWestern AustraliaAustralia; ^4^ Department of Pathology and Laboratory MedicineUniversity of Western AustraliaPerthWestern AustraliaAustralia; ^5^ Cell & Tissue Therapies Western Australia, Royal Perth HospitalPerthWestern AustraliaAustralia

**Keywords:** Lung transplantation, Graft rejection, Cell‐ and tissue‐based therapy, Mesenchymal stromal cells, Clinical trial, Phase 1

## Abstract

Chronic lung transplant rejection (termed chronic lung allograft dysfunction [CLAD]) is the main impediment to long‐term survival after lung transplantation. Bone marrow‐derived mesenchymal stromal cells (MSCs) represent an attractive cell therapy in inflammatory diseases, including organ rejection, given their relative immune privilege and immunosuppressive and tolerogenic properties. Preclinical studies in models of obliterative bronchiolitis and human trials in graft versus host disease and renal transplantation suggest potential efficacy in CLAD. The purpose of this phase 1, single‐arm study was to explore the feasibility and safety of intravenous delivery of allogeneic MSCs to patients with advanced CLAD. MSCs from unrelated donors were isolated from bone marrow, expanded and cryopreserved in a GMP‐compliant facility. Patients had deteriorating CLAD and were bronchiolitis obliterans (BOS) grade ≥ 2 or grade 1 with risk factors for rapid progression. MSCs (2 x 10^6^ cells per kilogram patient weight) were infused via a peripheral vein twice weekly for 2 weeks, with 52 weeks follow‐up. Ten Patients (5 male, 8 bilateral, median [interquartile range] age 40 [30–59] years, 3 BOS2, 7 BOS3) participated. MSC treatment was well tolerated with all patients receiving the full dosing schedule without any procedure‐related serious adverse events. The rate of decline in forced expiratory volume in one second slowed after the MSC infusions (120 ml/month preinfusion vs. 30 ml/month postinfusion, *p* = .08). Two patients died at 152 and 270 days post‐MSC treatment, both from progressive CLAD. In conclusion, infusion of allogeneic bone marrow‐derived MSCs is feasible and safe even in patients with advanced CLAD. Stem Cells Translational Medicine *2017;6:1152–1157*

**Significance Statement**

Long‐term survival after lung transplantation is compromised by the development of chronic lung allograft dysfunction (CLAD) which is characterized by inflammation, fibrosis, respiratory failure, and death. Ten‐year post‐transplant survival is only 30%‐40%, with CLAD explaining much of this mortality. Preclinical studies suggest that mesenchymal stromal cell (MSC) treatment will be effective in CLAD. However, safety concerns remain, since MSCs have the capacity to differentiate into pro‐fibrotic cells, potentially implicating them in graft fibrogenesis. In this first‐in‐man study, we demonstrate that MSC therapy is feasible and safe in patients with CLAD, providing an important foundation for future studies to assess efficacy.

## Introduction

Long‐term survival after lung transplantation is compromised by the almost inevitable development of chronic lung allograft dysfunction (CLAD) which results from recurrent and compounding alloimmune, infectious and other insults and is characterized by neutrophilic inflammation, fibrosis, respiratory failure, and death. Ten‐year survival following transplantation is only 30%‐40%, with CLAD explaining much of this mortality, and has changed little over three decades 1.

Bone‐marrow derived mesenchymal stromal cells (MSCs) hold great promise in the fields of allogeneic solid organ and bone‐marrow transplantation since they are able to abrogate T‐cell mediated immune responses 2, foster long‐lasting peripheral tolerance through the induction of a regulatory phenotype in CD4^+^ lymphocytes 3, 4 and attenuate neutrophilic inflammation through the secretion of tumor necrosis factor‐α‐stimulated gene 6 (TSG6) in response to pro‐inflammatory stimuli 5. These favorable characteristics have recently been translated into proven efficacy in human renal transplantation 6. Several preclinical studies now suggest that MSC treatment will be effective in CLAD 7, 8, 9, 10. However, despite these studies, safety concerns remain since MSCs have the capacity to differentiate into fibroblast–like cells and were found in bronchoalveolar lavage fluid obtained from patients with CLAD, potentially implicating them in the pathogenesis of allograft fibrosis 11. Furthermore, MSCs are potently immunosuppressive, possibly increasing the risk of infection.

The primary objective of this study was to assess the feasibility and safety of intravenous delivery of allogeneic, human leukocyte antigen‐unmatched, bone‐marrow derived MSCs in patients with advanced CLAD. Our secondary objectives were to document changes in forced expiratory volume in one second (FEV_1_) and 6‐minute walk distance (6MWD) after MSC infusion, and to document survival at 12 months.

## Materials and Methods

This was a phase I, open‐label, dual‐center, nonrandomized evaluation of subjects diagnosed with CLAD. Subjects received MSC infusions, at a dose of 2 × 10^6^ cells per kilogram of bodyweight for each infusion, twice weekly for 2 weeks. Up to 10 subjects who met all eligibility criteria and who provided written informed consent were planned to be studied, with an interim review of safety by an independent data safety monitoring board after the first four patients had been treated, prior to recruitment of the final six patients. The two sites in Brisbane, Queensland and Perth, Western Australia, share very similar immunosuppression and post‐transplant care protocols, with the use of basiliximab induction and tacrolimus, mycophenolate and prednisolone based immunosuppression.

### Patients

Transplant recipients with single, bilateral, or heart‐lung allografts and BOS grade 2 or 3, or BOS grade 1 with an additional risk factor for poor outcome at our center (single lung transplant, rapid deterioration (>20% fall in FEV_1_ in the previous 12 months), or a pretransplant diagnosis of idiopathic pulmonary fibrosis or pulmonary hypertension) were potentially eligible. BOS was diagnosed and graded according to the 2002 iteration of the ISHLT guidelines 12. Only patients with progressive disease (defined as deteriorating FEV_1_ and/or worsening BOS grade) within the last 12 months were eligible. Patients had to have had stable immunosuppression doses and levels for 6 weeks prior to enrollment, and all received azithromycin in an attempt to treat CLAD. Patients were excluded if they had any of the following: active infection, acute allograft rejection, airway anastomotic complications, > 3 infective exacerbations of BOS in the last 12 months, a history of cytomegalovirus pneumonitis, poor functional status not expected to survive 3 months, pregnancy or breastfeeding or an allergy to beef products. All patients provided written informed consent prior to the commencement of any study‐related procedures. The study (www.clinicaltrials.gov NCT01175655) was approved by The Prince Charles Hospital and Royal Perth Hospital Human Research and Ethics Committees.

### MSCs

MSCs were produced under good manufacturing practice conditions (Therapeutic Goods Administration Licence No: 44165) from five unrelated donors (3 female, aged 17–30). MSCs were not pooled, and each recipient only received MSCs from one donor. In brief, 10 ml of bone marrow was aspirated from unrelated donors medically assessed as suitable and serologically negative for HIV‐1/‐2, HCV, HBV, HTLV‐1/‐2, and syphilis. MSCs were isolated from the mononuclear fraction and culture expanded up to passage 5. Release criteria included viability > 70% (trypan blue), negative microbial contamination testing and a typical MSC immunophenotype (CD105^+^CD90^+^CD73^+^CD45^−^) and trilineage differentiation capacity 13. Cytogenetic testing was performed on final product MSCs by a NATA accredited laboratory. Cells were cultured to >65% confluence and metaphases obtained after exposure to colchicine. A minimum of 15 metaphases were fully analyzed by G banding. MSCs were cryopreserved in 10% dimethyl sulphoxide, 50% Plasmalyte (Baxter, Sydney, http://www.baxterhealthcare.com.au/), 20% normal saline and 20% human serum albumin in doses of 50 and 100 x 10^6^ cells and stored below −150°C. Cryopreserved MSCs were transported to the trial centers in monitored liquid nitrogen dry shippers and stored at <−150°C until use.

### Infusion and Follow‐Up

MSCs (2 × 10^6^/kg for each infusion, infused twice weekly for 2 weeks) were thawed in a water bath, diluted 1:1 with Plasmalyte (Baxter), and infused via gravity feed into a large peripheral vein over 15 minutes. Observations (heart rate, blood pressure, peripheral oxygen saturation) were made at baseline and at 15, 30, 45, 60 minutes and hourly until 4 hours after infusion. Patients were followed up at 1, 2, 3, 4, and 6 weeks and at 2, 3, 6, and 12 months postinfusion with blood toxicology, chest x‐ray, and spirometry (performed as per American Thoracic Society guidelines 14). 6MWD was assessed at 1, 3, 6, and 12 months again according to ATS guidelines 15.

### Statistics

Data are presented as median (interquartile range) unless otherwise stated. Pre‐ and post‐MSC infusion data was assessed using Wilcoxon matched‐pairs signed‐ranks test with *p* < .05 used to define statistically significant differences.

## Results

**Figure 1:**
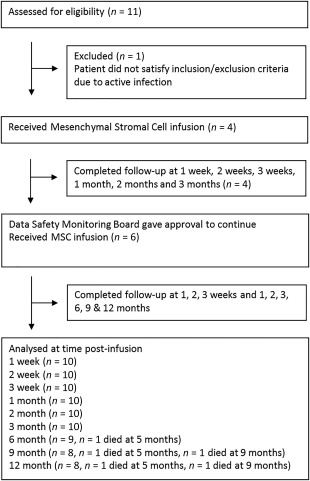
Clinical trial flow diagram. Abbreviation: MSCs, mesenchymal stromal cells.

Eleven patients were screened for inclusion. One patient was excluded due to active infection (Fig. 1), leaving 10 patients who received MSCs. All patients received the full dosing schedule in the outpatient setting. No patients were lost to follow‐up although some 6MWD data was missing. Patient demographics are provided in Table 1. All patients were unresponsive to azithromycin.

**Table 1:** Baseline demographics

	Cohort (*n* = 10)
Age, years, median (IQR)	37.1 (26.2–51.5)
Male sex, *n* (%)	5 (50)
Transplant type, *n* (%)	
Single	2 (20)
Bilateral	8 (80)
Pretransplant diagnosis, *n* (%)	
Cystic fibrosis	4 (40)
Chronic obstructive pulmonary disease	2 (20)
Idiopathic pulmonary fibrosis	3 (30)
other	1 (10)
BOS grade at infusion, *n* (%)	
BOS2	3 (30)
BOS3	7 (70)
FEV_1_ (l), median (IQR)	1.2 (1.0–1.3)
6MWD (min), median (IQR)	472.5 (317.5–617.0)

Abbreviations: 6MWD: 6‐minute walking distance; BOS: bronchiolitis obliterans syndrome; FEV_1_: forced expiration volume in 1 second; IQR: interquartile range.

**Figure 2:**
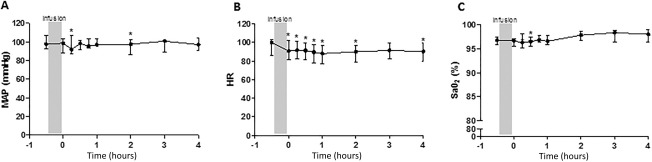
Effect of mesenchymal stromal cell (MSC) treatment on hemodynamics and gas exchange. **(A)**: MAP, **(B)**: HR, and **(C)**: SaO_2_ following MSC infusion. Data are presented at median ± interquartile range, *, *p* < .05 versus preinfusion. Abbreviations: HR, heart rate; MAP, mean systemic arterial pressure; SaO_2_, peripheral oxygen saturation.

There were no serious adverse events attributable to MSC therapy. For the total 40 MSC infusions which were administered, the most common adverse events felt by the investigators to be possibly or probably related to the MSC infusion were halitosis (12 episodes), liver function test abnormalities (3), lower respiratory tract infection symptoms (3) and dizziness (2). Other reported adverse events, each with a frequency of one were: headache, raised white cell count, raised lactate, raised platelet count, nausea, vomiting, ascites, cholecystitis, insomnia, and somnambulism. Grouped data revealed a small fall in mean arterial pressure and oxygen saturation within 30 minutes of the MSC infusion (Fig. 2), but with rapid recovery. However, there were no individually reported adverse hemodynamic or gas exchange events. There were no adverse toxicological signals. Two patients died, both from progressive BOS at 152 and 270 days after the final MSC infusion. Neither death was felt to be related to MSC treatment.

**Figure 3:**
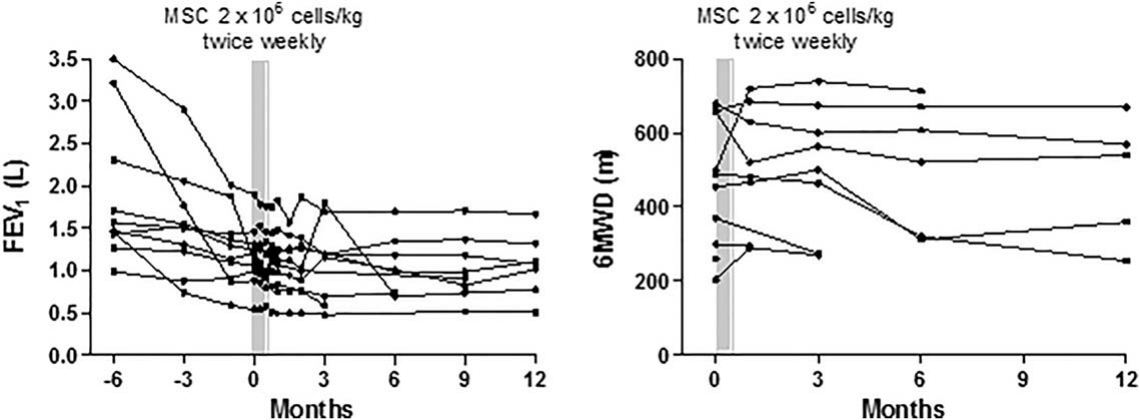
Effect of mesenchymal stromal cell (MSC) treatment on lung function and walk distance. Lung function **(A)** and 6MWD **(B)** before and after intravenous infusion of MSCs. Abbreviations: 6MWD, 6‐minute walking distance; FEV_1_, forced expiration volume in 1 second; MSCs, mesenchymal stromal cells.

The rate of FEV_1_ decline slowed, but this was not statistically significant (from 120 ml/month to 30 ml/month after MSC treatment, *p* = .08; Fig. 3A). Assessment of the effect of MSC treatment on 6MWD was hampered due to the number of missing values (Fig. 3B). There was no evidence of new onset or worsening of interstitial fibrosis on interval chest x‐rays.

## Discussion

CLAD remains the major impediment to long‐term survival after lung transplantation, and has proven frustratingly refractory to attempts at treatment, apart from an apparent effect of azithromycin in some patients 16. CLAD can be considered as the end‐result of recurrent and compounding episodes of graft injury, most of which are alloimmune in nature, although nonspecific (e.g., gastro‐oesophageal reflux disease, infection) and autoimmune injuries no doubt also play a role. Approaching alloimmune injury with further immunosuppression has proven counter‐productive with patients suffering an increased burden of infections without altering the natural history of allograft failure. An alternative approach, whereby partial tolerance to graft antigens is induced, so that immunosuppression can be stabilized or even reduced, is thus an attractive approach to CLAD management. In this study, we confirmed the feasibility and safety of one such approach—intravenous delivery of allogeneic MSCs—laying the foundation for future studies to assess efficacy.

When planning this study, our main safety considerations were previous reports suggesting that a putative lung MSC could be profibrotic 11, and the potential for hemodynamic or gas exchange compromise following embolization of intravenously delivered cells to a compromised pulmonary vascular bed. However, we saw no evidence for worsening fibrosis in this study, or in a previous study of intravenous delivery of MSCs in idiopathic pulmonary fibrosis 17. These two studies in patients with severe fibrotic lung disease add to the large body of safety data for the intravenous administration of MSCs to humans with other diseases. The multifaceted activity of MSCs led to very significant clinical trial activity outside the lung, most notably in the treatment of steroid refractory graft versus host disease following allogeneic bone marrow transplant, but also in other immune‐mediated diseases like Crohn's disease, multiple sclerosis, lupus and in the renal transplant setting 6. Many thousands of patients have now received MSCs via intravenous infusion for these indications with few adverse events noted 18.

MSCs are a specialized stromal cell type, originally identified in suspensions of bone marrow and spleen 19. MSCs or MSC‐like cells have now been identified in many organs, including the lung 20. They are characterized by their tendency to adhere to plastic (this remains the predominant means of isolation); their ability to form colonies from single cells when plated ex vivo at clonogenic levels; their fibroblast‐like appearance; their multipotent (fat, cartilage, and bone) differentiation capacity; and their surface immunophenotype. MSCs are poorly immunogenic, can escape lysis by cytotoxic T cells and natural killer cells and so can be transplanted between HLA‐mismatched individuals without the need for immunosuppression. Although this relative immune privilege has been reported in multiple studies using xenogeneic and major histocompatibility mismatched models, it is not total 21, so that delivery of alloegeneic cells to an immunosuppressed host, as was done in this study, may be an advantage.

As a stem cell, it was originally thought that MSCs may be able to act as a convenient agent of organ regeneration. Although MSCs are able to differentiate, under stringent and artificial laboratory conditions, to multiple cell types including lung epithelium, it is now clear that engraftment in target tissues and such transdifferentiation occurs minimally and does not explain the significant therapeutic benefit shown in multiple studies of inflammatory conditions. MSCs have to some extent hence been repurposed for use as immunosuppressive and immunomodulatory agents by teams interested in their therapeutic use. These anti‐inflammatory properties are curious in a cell type which largely provides stromal support to tissues and is an integral part of the stem‐cell niche(s) in multiple organs, but may in fact be directly related to this “stemness.” In order to persist in tissues such as lung for long periods (a key feature of stem cells), the usual cues which initiate apoptosis—in particular the accumulation of dysfunctional mitochondria—need to be overcome. MSCs have evolved the ability to “outsource” the removal of dysfunctional mitochondria (a process termed mitophagy) to macrophages in order to suppress apoptotic cues, and have coevolved powerful immunosuppressive and immunomodulatory signaling mechanisms to suppress the overwhelming inflammatory response which would otherwise inevitably follow the potent damage signal of macrophage‐mediated mitophagy 22. Hence, although the stem‐like and anti‐inflammatory properties of MSCs are linked through mitophagy, it is the anti‐inflammatory properties which have led to MSCs being studied to treat inflammatory and immune‐mediated diseases. Since the pro‐inflammatory macrophage phenotype has been implicated in the pathogenesis of obliterative bronchiolitis 23, 24, the idea behind the work presented here is to use this feature of MSC biology to favorably alter macrophage phenotype and CLAD natural history, an approach previously found to be effective in preclinical studies 9, 10.

The “first‐pass effect” whereby cells delivered intravenously are required to transit the lung so that there is extensive and homogeneous, although admittedly temporary, retention as they pass through the pulmonary circulation is a major advantage for cell therapies designed to treat pulmonary disease as delivery is relatively straightforward. Even more attractively, retained cells target areas of injured/inflamed lung, remain for up to 7 days and are activated upon reaching the target site to secrete prostaglandin‐E_2_ (PGE_2_) and TSG6 25, 26. In support of this idea, we found that culture of bone‐marrow derived MSCs in media supplemented with BAL supernatant obtained from CLAD affected lungs led to significant upregulation of TSG6 expression (data not shown). It is TSG6 which mediates the anti‐inflammatory effect of MSCs retained in the lung on macrophages 27 and other cells 28, even in organs as distant as the cornea and heart 29. Evasion of the host immune system is also in part TSG6 dependent 30, as is the induction of Treg 3. Finally, TSG6 also directly inhibits neutrophil migration by binding IL8 31, and explains most of the therapeutic effect in acute lung injury and bleomycin‐induced pulmonary fibrosis 32. In addition to their immune suppressive properties, MSCs secrete a variety of anti‐fibrogenic proteins and enzymes such as interleukin‐10 (IL‐10), hepatocyte growth factor and matrix metalloproteinases and are effective in bleomycin‐induced lung fibrosis 33. These properties, in combination with targeted and persistent TSG6, PGE_2_ and IL‐10 secretion by MSCs retained and activated in areas of abnormal lung, underlie their therapeutic effect in inflammatory diseases and animal models of transplantation 34, and point to MSC therapy potentially providing a “magic bullet” to treat CLAD.

Several limitations to the work presented here need to be acknowledged. First of all our data are uncontrolled, so we are unable to comment on efficacy. It is known that the natural history of CLAD is for the rate of lung function decline to sometimes taper as disease progresses—akin to the “ground effect” experienced by pilots as airplanes come in to land. Nevertheless, the difference in rate of decline observed before and after MSC treatment (120 vs. 30 ml/month) is similar to that observed in previous retrospective studies of extracorporeal photophoresis (116 vs. 28.9 ml/month) 35 and total lymphoid irradiation (122.7 vs. 25.1 ml/month) 36 for BOS in which the authors felt the treatments had been effective. Second, we did not systematically obtain ancillary clinical material (e.g., peripheral blood and bronchoalveolar lavage fluid) to investigate possible mechanisms. This will be a key objective in future studies. Furthermore, although patients in this study had advanced CLAD, it is likely that therapies designed to prevent CLAD progression will be more effective if delivered earlier in the disease course, potentially even before CLAD develops. Finally, in this study patients received an initial salvo of four infusions, with no further infusions after baseline. Although sustained tolerogenic effects have been noted following MSC infusion 4, it is likely that future MSC‐based treatments will require a repeated dosing schedule. The timing of such repeat treatments is not currently known, and will only be able to be determined when mechanism and duration of therapeutic effect are assessed in larger, controlled studies. To this end, we have recently secured funding to conduct a phase 2 study in Australia to assess the efficacy of MSC therapy in CLAD. The ASSIST‐CLAD (Australian Study of Stem Cell Therapy to Induce Sustained Tolerance in patients with CLAD, NCT02709343) study will randomize 82 patients with new onset CLAD to intravenous bone marrow‐derived MSC therapy or placebo in a 1:1 ratio.

## Conclusion

In this first‐in‐man study, we have demonstrated that intravenous MSC therapy is feasible and safe in patients with CLAD, providing an important foundation for the conduct of future controlled studies to assess efficacy.

## Author Contributions

D.C.C. and P.M.A.H.: conception and design, provision of study material or patients, collection and/or assembly of data, data analysis and interpretation, manuscript writing, final approval of manuscript; D.E.: administrative support, collection and/or assembly of data, data analysis and interpretation, final approval of manuscript; S.L.: administrative support, provision of study material or patients, collection and/or assembly of data, final approval of manuscript; M.J.S.: conception and design, provision of study material or patients, manuscript writing, final approval of manuscript; R.H.: provision of study material or patients; final approval of manuscript; S.Y.: conception and design, administrative support, data analysis and interpretation, manuscript writing, final approval of manuscript; M.M.: conception and design, provision of study material or patients, final approval of manuscript.

## Disclosure of Potential Conflicts of Interest

M.S. is a Director of Isopogen which has licensed MSC manufacturing to Cell and Tissue Therapies WA. None of the other authors have a potential conflict of interest.
